# Roles of Transposable Elements in the Different Layers of Gene Expression Regulation

**DOI:** 10.3390/ijms20225755

**Published:** 2019-11-15

**Authors:** Denise Drongitis, Francesco Aniello, Laura Fucci, Aldo Donizetti

**Affiliations:** 1 Institute of Genetics and Biophysics “Adriano Buzzati Traverso”, Consiglio Nazionale delle Ricerche, 80131 Naples, Italy; denise.drongitis@igb.cnr.it; 2 Department of Biology, University of Naples Federico II, 80126 Naples, Italy; faniello@unina.it (F.A.); fucci@unina.it (L.F.)

**Keywords:** transposable elements, gene expression regulation, TEs co-option

## Abstract

The biology of transposable elements (TEs) is a fascinating and complex field of investigation. TEs represent a substantial fraction of many eukaryotic genomes and can influence many aspects of DNA function that range from the evolution of genetic information to duplication, stability, and gene expression. Their ability to move inside the genome has been largely recognized as a double-edged sword, as both useful and deleterious effects can result. A fundamental role has been played by the evolution of the molecular processes needed to properly control the expression of TEs. Today, we are far removed from the original reductive vision of TEs as “junk DNA”, and are more convinced that TEs represent an essential element in the regulation of gene expression. In this review, we summarize some of the more recent findings, mainly in the animal kingdom, concerning the active roles that TEs play at every level of gene expression regulation, including chromatin modification, splicing, and protein translation.

## 1. Introduction

The mobile genetic elements known as transposable elements (TEs) were discovered in the late 1940s thanks to Barbara McClintock’s [1,2] seminal work on maize, a discovery that revolutionized the previous view of genes as stable entities on chromosomes. TEs are an important driving force in genome evolution, and bursts of TEs have been connected with significant evolutionary events such as the speciation process [3]. TEs are DNA fragments that have the ability to mobilize within a host genome, often creating new copies of themselves during the mobilization process.

### 1.1. Transposable Elements Classification

A detailed classification has been extensively reviewed elsewhere [4]. Briefly, TEs are divided into two large classes called Class I retrotransposons and Class II DNA transposons, which differ from each other in their mobilization mechanisms. Class I retrotransposons are transcribed into an RNA intermediate that can then be reverse transcribed into DNA and reintegrated as an additional copy in the genome. In contrast, Class II DNA transposons encode a transposase enzyme that cuts the parental sequence from the genomic sequence and then mediates its reintegration into another location in the genome [4,5]. Class I retrotransposons are further subdivided into five orders depending on their mechanistic features, organization, and reverse transcriptase phylogeny [4]. Class I includes long terminal repeats (LTR) retrotransposons, such as the endogenous retroviruses (ERVs) superfamily, and long and short interspersed nuclear elements (LINEs and SINEs, respectively) [4,6,7]. Class II DNA transposons can be divided into two subclasses on the basis of the number of DNA strand that are cut during transposition, and includes the order of terminal inverted repeat (TIR) transposons in the subclass I [4].

### 1.2. The Deleterious Effects of the Transposable Elements Mobilization

Many elements cooperate in TE integration such as the genomic sequence, chromatin, and nuclear contexts, which accounts for the diversity in insertion-site distribution and evolutionary strategies [8]. The ability of TEs to move through the genome can be detrimental to the host if not properly controlled. The deleterious effects of mobile element activation had long been assumed to be harmful by causing chromosome breakage or acting as a mutagen when targeted to protein-coding genes [9]. However, TE transposition can also occur during germline development and, less frequently, in somatic cells [10,11]. The deleterious effects of germline transpositions have been observed for some time, with different examples coming from different organisms and different transposon classes. For example, LINE1 (L1; Class I) insertions in human haemophilia A and the *P*-element DNA transposon (Class II) in *Drosophila* that is the basis for dysgenic traits [12,13]. Somatic transpositions have also been receiving much attention, as a growing body of evidence is accumulating that associates detrimental biological effects with the somatic transposition of TE. One of the most interesting examples of this is represented by the mariner-Mos1 element of Drosophila (Class II transposon), which can be transposed during the Drosophila life cycle and negatively affects behavioral activities and embryonic viability [14]. Probably one of the most characterized pathological states linked to a new wave of transpositions in somatic cells is cancer, including ovarian [15], colorectal [16], and Fanconi anemia [17] cancer, to name just a few. Transposable elements can escape from their epigenetic silencing, as has been shown in most cancers, where lower methylation levels (hypomethylation) and the dysregulated chromatin modification of L1 retrotransposons results in their integration into novel sites (insertional mutagenesis), a process that has been reported for esophageal squamous cell carcinoma [18] and pancreatic ductal adenocarcinoma [19]. In addition to cancer, it has become clear that TEs are implicated in the rise of different brain disorders, including schizophrenia [20], and autism [21]. In schizophrenia patients, whole-genome sequencing has revealed specific L1 insertions in the brain, probably triggered by environmental and/or genetic risk factors and, preferentially localized to synapse- and schizophrenia-related genes, this has led to the hypothesis that the hyperactivation of L1 retrotransposons may contribute to the susceptibility and pathophysiology of schizophrenia [22]. The aberrant activation and mobilization of TEs have also been associated with the pathophysiology of neurodegenerative diseases such as Alzheimer disease (AD). Guo et al. [23] have shown an interesting association between the differential expression of several TEs and the neurofibrillary tangle burden in post-mortem human brains, indicating an association between TE activation and genomic instability in Tau-mediated AD mechanisms. In addition, the silencing of TEs mobilization through epigenetic mechanisms has been hypothesized as a neuroprotective mechanism of cortical spreading depression (CSD), which is an evolutionarily conserved phenomenon that involves a slow, self-propagating depolarization wave that is associated with the spontaneous depression of electrical neuronal activity, which can promote neuroprotection by inducing preconditioning [24]. The effects of the epigenetic control of LINE sequence silencing by DNA methylation together with the histone modifications in CSD-induced tolerance might counteract genome destabilization, thus preventing disabling phenomena such as senescence [25,26].

### 1.3. Mechanisms to Control the Transposable Elements Mobilization

Organisms evolved into different mechanisms to control the mobilization of TEs in order to counteract the damages caused by their transposition. One well-known mechanism in germline is based on interactions between the TEs and a plethora of non-coding RNAs. The PIWI-interacting RNAs (piRNAs), which interact with TEs at numerous levels, are the most studied of the regulatory RNAs. These RNAs can induce silencing at the transcriptional level or cause epigenetic modifications that alter the protein accessibility to DNA required for transcription. At the post-transcriptional level, they can cause TE transcript degradation through the formation of double-stranded RNAs (dsRNAs) that can be cleaved into small-interfering RNAs (siRNAs), and they are associated with the RNA-induced silencing complex (RISC). In general, piRNAs act in the gonads to protect male and female germ-line genomes from transposable elements. Through a mechanism conserved among different species, piRNAs repress TE expression by modifying the histone 3 lysine 9 (H3K9) methylation state [27]. Specifically, nascent transcripts derived from full-length LINEs are recognized by MIWI2 protein/piRNAs complex that recruits a histone methyltransferase, resulting in the deposition of the histone 3 trimethyl lysine 9 (H3K9me3) mark on LINE repeats in the germ-line genome [27]. In an ovarian somatic cell line, piRNAs also formed complexes with H1/H3K9me3 and heterochromatin protein 1a (HP1a), modulating chromatin accessibility and thus affecting TE transcription [28]. There is also evidence for the piRNA-induced silencing of TEs in *Drosophila* somatic cells [29]. Other molecular mechanisms have also evolved, such as the distribution of TEs in regions of low gene density and the packaging of TEs into transcriptionally silent heterochromatin [30]. DNA methylation has largely been considered a strategy for blocking transposon mobility and preserving genome integrity in a wide range of cells, tissues, organisms, and biological contexts. The strict relationship between TEs and their silencing by DNA methylation is reflected by the fact that, because TEs represent such a large fraction of the mammalian genome, TE methylation analyses are often used as surrogates for global DNA methylation analyses [31,32].

## 2. Transposable Elements as an Essential Part of Gene Expression Regulation

TE mobilization, expression, and insertion can be deleterious if not property controlled, with negative effects on cell physiology. However, at the same time, they can be normally involved in shaping genomic structure and controlling gene expression. This is, for instance, evident in adult brain tissue, where a pervasive mobilization of the mobilization of the L1, Alu, and SINE-R/VNTR/Alu (SVA) transposons can reshape the gene regulatory networks of different kinds of neurons, particularly those in the hippocampus [33,34]. Similarly, other studies have also indicated a functional role for L1 transposition in neuronal precursor cells [11,35]. The expression of TEs in the brain is emblematic of this “double-edged sword” phenomenon, given that TEs can play a dual and conflicting role in the correct differentiation and development of neural mosaicism and in the onset of neurological disease. These are interesting examples of how TE evolution includes not only mechanisms for silencing the deleterious effects of these invading sequences, but also mechanisms for them to take an active and positive role in genome function. It is becoming clear that TEs are an integral part of the regulatory toolkit of the genome [36]. Repetitive elements have emerged as potential regulators for various biological processes [37], such as gene transcription, alternative splicing, and RNA translation. Even more evidence has accumulated showing how TEs can be instrumentally involved in the control of gene expression.

### 2.1. Transposable Elements and the Epigenetic Regulation of Gene Expression

Historically, the relationship between TEs and epigenetic modifications has often been seen as consisting of silencing TEs by DNA methylation and histone modification, with the distribution of TEs seemingly having evolved in parallel with the strategies to control their expression [38]. However, transposable elements are also emerging as an important source of epigenetic marks that can influence gene expression, leading to the idea that TE insertion may be involved in targeting epigenetic modifications to a specific locus. The causal effect of TE mobilization and distribution on the epigenetic regulation of gene expression is still a complex and unresolved issue. Evolution has favored that active intragenic TEs be preferentially inserted in an antisense direction with respect to the genes, producing a “trap” mechanism that can stop the invasion of these sequences during demethylation waves along the genome. To silence the TEs, these overlapping sense/antisense transcripts enter an endo-siRNA pathway that is regulated by DICER and Argonaute 2 (AGO2) and, when activated by global demethylation, leads to an increase in the repressive histone marks [39]. The molecular pathway that underlies this link between the repression of TEs and the increase in histone repressive marks remains yet to be revealed [39]. New research in naïve murine embryonic stem cells pre-implantation indicates a central role for the KZFP/KAP1 (Krüppel associated box (KRAB) zinc finger protein/KRAB-associated protein 1) complex in maintaining heterochromatin, with DNA methylation marks at TEs protecting the loci from ten eleven translocation (TET)-mediated demethylation [40]. Ecco et al. [41] found that in differentiated tissues, two members of the KRAB/ZFP (KRAB/Zinc Finger Protein) family regulate TE targets through histone-based mechanisms that are not always correlated with the DNA methylation status of the loci. It is clear from the study that the interactions between the TEs and their KRAB-ZFP controllers influence nearby gene expression. This interaction is even clearer in human neural progenitor cells where it has been demonstrated that primate-specific ERVs act as docking platforms for the co-repressor protein KAP1 (also known as TRIM28) to establish local heterochromatin [42]. KAP1 binds and represses the ERVs, and consequently, regulates the expression of neighboring genes important for brain development [42]. Another example of the regulation of neighbouring genes by TEs includes the interactions of the transcriptional regulators human silencing hub (HUSH) and microrchidia family CW-type zinc finger 2 (MORC2) with evolutionarily young full-length L1s located in the transcriptionally permissive euchromatic region, which promotes the deposition of histone H3K9me3, a specific mark for transcriptional silencing. This MORC2/HUSH-bound L1 specific effect can spread to the neighboring genes, inducing a decrease in mRNA expression and probably influencing the RNA polymerase II (POL II) elongation rate [43]. Being a source of chromatin variation, specific classes of TEs (especially younger LINEs) have been found to impact chromatin accessibility in the livers of different inbred mice strains. This shows that TEs can regulate tissue-specific genes, which could result in downstream phenotypic diversity among populations [44]. Transposable elements can also exert a long-range regulation of gene expression by actively reshaping chromatin organization. Generally, and across different tissues, about 10% of TE families have been found to be enriched in active genomic regions. Among these, SINEs and DNA transposons are the most frequently enriched classes in the active chromatin regions, while L1 LINEs and ERV LTRs are the most frequently enriched TE classes in the repressed regions targeted with the H3K9me3 epigenetic mark [45].

#### Mechanisms of the Transposable Elements Co-option in the Epigenetic Control of Gene Expression

**Figure 1:**
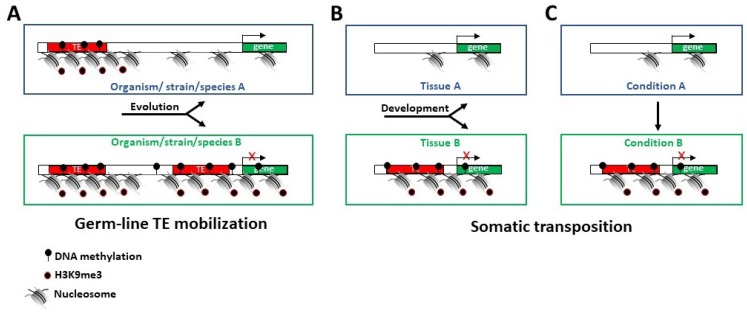
Effect of TE localization on the epigenetic regulation of gene expression. (**A**) The different distribution of TEs during evolution reshapes the epigenetic regulation of a specific gene. (**B**) The differential redistribution of TEs in different cells/tissues of the same organism during the development affects the expression of a specific gene. (**C**) The relocalization of TEs sequence in the same cell after a specific stimulus/condition affects the expression of a specific gene.

Intriguingly, the epigenetic influence of TEs is particularly evident in open euchromatin regions. For instance, the enrichment of repressive epigenetic marks around euchromatic TEs is due to the presence of TEs, rather than due to the preferential insertion of TEs into genomic regions already enriched with repressive epigenetic marks, as has been demonstrated by comparing the epigenomes of two *D. melanogaster* strains. This distribution accounted for finding higher histone 3 dimethyl lysine 9 (H3K9me2) enrichment and lower transcript levels for TE-flanking alleles than for the homologous alleles lacking nearby TE insertions [46]. Similarly, the analysis of epigenetic marks in flies with and without *Bari-Jheh*, a natural transposon that affects the expression of nearby genes, showed significant differences in histone 3 trimethyl lysine 4 (H3K4me3), H3K9me3, and histone 3 trimethyl lysine 27 (H3K27me3) histone marking in relation to oxidative stress conditions, highlighting that this TE element influences gene expression by affecting the local chromatin state [47]. These examples suggest that the different distribution of TEs occurring in the germlines of different organisms/strains and species strongly affect the gene expression of nearby genes (Figure 1A).

Interestingly, different TE distributions due to somatic transpositions affecting gene expression are also relevant in the same individual, leading to the idea that TEs have been co-opted, and their distributions co-evolved with gene expression regulation [48]. In fact, TE enrichment varies across tissues and TEs contain binding sites for tissue-specific master regulators of transcription [45]. One element of TE targeting comes directly from the fact that integration is restricted to open chromatin regions. This is apparent in mammalian brains, where somatic LINE insertions are enriched in the proximity of neuronal genes [11,12,13,14,15,16,17,18,19,20,21,22,23,24,25,26,27,28,29,30,31,32,33]. If we consider that TEs can be the target of epigenetic marks, the non-random and targeted tissue-specific distribution of TEs can be considered a mechanism to genetically fix a landmark, which could induce an epigenetic control of nearby gene expression (Figure 1B). The finding that different environmental factors induce L1 transposition through different basic helix-loop-helix PER-ARNT-SIM (bHLH/PAS) proteins opens the possibility of the differential targeting of L1 insertions during different types of stresses [49]. Experimental findings hint that TE insertions are targeted beyond the apparent mechanistic necessity of open chromatin [50]. In *Arabidopsis thaliana*, TEs located in proximity to environmentally induced genes are silenced via enhanced DNA methylation, establishing a temporal and functional hierarchy of transcriptional and epigenomic changes in response to stress [51]. It is possible to argue that the mobilization and insertion of TEs can also be controlled in adult tissues and post-mitotic cells after specific stimuli for the purpose of driving the epigenetic regulation of specific genes (Figure 1C).

### 2.2. Transposable Elements and Long-Range Regulation

Throughout evolution, different families of TEs have originated multiple binding sites for transcription factors, producing different transcriptome landscapes. The ENCODE (Encyclopedia Of DNA Elements) data contains about 2 million transcription factor binding sites (TFBSs) that overlap with putatively regulation-competent human retrotransposons, which are located in a 5-kb gene promoter neighborhood composed of ~44% SINEs, ~33% LINEs, and 23% LR/ERVs [52]. The data show that LINE-derived transcription factor binding sites (TFBSs) are more numerous than SINEs outside the 5-kb region near the transcription start site, while the reverse occurs within the region. Moreover, the pathways most strongly impacted by the different retrotransposon distributions have been implicated in important processes, such as the cell stress and immune responses, ribosome biogenesis, chromatin remodeling, and DNA replication as well as mitotic spindle organization and cell cycle progression [52]. Nishihara et al. [53] were the first to report that an evolutionarily conserved genomic region named AS3_9, comprised of three TEs inserted side-by-side, functions as a distal enhancer for *wnt5a* expression during the morphogenesis of the mammalian secondary palate. Functional analyses have shown that, during mammalian evolution, the AmnSINE1, X6b_DNA, and MER117 retrotransposons have been co-opted together by a retroposition/transposition mechanism, with the acquisition inside of X6b_DNA sequence of a specific binding site for Msx1 protein, which together with Wnt5a is involved in the palatogenesis. This study suggested that the combination of different TEs located within the same DNA fragment could have played a significant role in the evolution of the high diversity of many *cis*-regulatory elements [53].

### 2.3. mRNA Decay and Splicing

**Figure 2:**
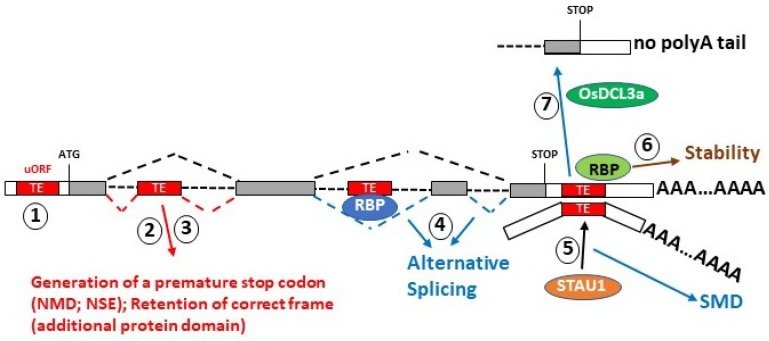
TE-dependent post-transcriptional regulation of gene expression. (1) TE inside the 5′-UTR cause the presence of an upstream ORF that participates in the regulation of the main ORF translation; (2) the exonization of a TE and thus its translation determines the presence of an additional domain inside the encoded protein; (3) the exonization of TE inside the coding region of an mRNA can result in the presence of a premature stop codon, thus resulting in Nonsense-mediated Decay process. (4) TE sequence can interact with RNA-binding proteins (RBP) and affects alternative splicing; (5) the presence of a TE sequence in the 3′-UTR of an mRNA can trigger STAU-mediated decay, (6) serves as a docking station for RBP involved in RNA stability such as HuR protein, (7) or triggers translational repression through the generation of a shorter mRNA without poly-A tail.

Transposable Elements can also be involved in gene expression regulation, affecting mRNA stability through non-sense mediated decay as well as the activity of microRNAs, circular RNAs, and potentially long non-coding RNAs (lncRNAs). The Alu sequences, a subfamily of SINEs elements, are often embedded in pre- and mature mRNAs, usually as a part of the 3′-untranslated region (UTRs) or introns [54]. The Alu elements contained in mRNA sequences (and lncRNAs) can play a role in Staufen-mediated decay (SMD). It has been reported that STAU1 binding sites can be formed by imperfect base pairing between an Alu element within the 3′-UTR of an SMD target and another Alu element within a cytoplasmic and polyadenylated lncRNA. These sites can engage the STAU protein, triggering SMD through a downregulated mRNA expression profile [55] (Figure 2).

Lineage-specific 3′-UTR SINEs can function to regulate the levels of mRNAs from orthologous genes in different species (including human) by directing SMD, demonstrating the specific role that lineage-specific SINEs have played in the convergence of gene expression profiles between species [56]. Transposable element-derived sequences localized in RNA transcripts can also play a role in the regulation of mRNA abundance and alternative splicing. In a process called exonization, TEs inserted into introns are recognized by the splicing machinery and recruited into RNA transcripts as exons. TEs contain numerous splice-donor and -acceptor sites that contribute to alternative splicing. Thus, they can interact with a plethora of RNA binding proteins (RBPs) with composition site preferences that drive them to specific regions of TEs, for example Human antigen R (HuR) or Fused in Sarcoma (FUS) proteins, that prefer to bind to U-rich motifs [56]. However, the depletion of TE binding sites for different RBPs indicates these sites affect transcript abundance and splicing in a way similar to that seen in gene-binding sites located in non-repetitive sequences [56]. It has been also noticed that in a few specific cases, the effect of RBP binding can change depending on the specific TE family bound. For example, the RBP HuR confers transcript stability unless it is bound to an Alu element in a U-rich region [57] (Figure 2). The Alu-containing RNAs may also be capable of forming stable structural domains, which would likely give rise to novel biological functions [58]. Kralovicova et al. [59] have shown that an intronic transposed element highly similar to medium reiterated frequency repeat family 51 (MER51A) can influence gene expression, modulating the inclusion levels of many nonsense-mediated RNA decay switch exon (NSEs), pseudo-exons derived by the activation of cryptic splice sites that act as a buffer to counteract Alu-mediated NSE activation (Figure 2).

### 2.4. Transposable Elements and Noncoding RNAs

Another class of small RNAs implicated in TE regulation is the circular RNAs (circRNA), which are a new class of small noncoding RNAs with gene regulation functions. Recent mammalian studies have shown that the flanking regions of circRNAs are enriched with transposons that have the potential to mediate circRNA formation via reverse complementary pairing [60,61]. In humans, the presence of different circRNAs derived from a single gene locus is associated with a variety of Alu pairings in human introns, suggesting a role for competition between the pairings in the formation of alternative circularizations [62]. A new study in maize that sequenced circRNA showed that LINE1-like elements and their reverse complementary Pairs (LLERCPs) are significantly enriched in the flanking regions of circRNAs [63]. Interestingly, when LLERCP transcription increases, the accumulation of circRNAs varies, leading to a decrease in linear transcripts [63]. Jung et al. [64] described how TEs can affect the expression of another class of long non-coding RNAs called cis-natural antisense transcript (cis-NATs) in different ways. First, NATs can originate from TEs and can be transcribed by alternative promoters depending on the TE. They can also be exonized by TEs, with the newly formed exon being complementary to the exon of a sense protein-coding gene [64]. Thus, the expression of sense transcripts may be mediated by NATs through dsRNA formations, that can participate in the RNA interference or adenosine deaminase acting on RNA (ADAR) pathways.

### 2.5. Transposable Elements and Protein Translation

Retrotransposons have evolved side by side with genes, inserting into different positions along the gene bodies and producing a plethora of effects. Transposable element insertions into 5′-UTRs or 3′-UTRs of mRNAs affects the protein expression of many genes in different ways (Figure 2). Kitano et al. [65] showed that TEs are also implicated in the upstream open reading frame (uORF)-mediated translational regulation of a large number of genes. The major families of retrotransposons, such as LINEs and SINEs can operate as cis-acting elements to inhibit or promote the translation of canonical ORFs located downstream of the uORFs in eukaryotic mRNAs. Using the human RefSeq mRNA sequence database, Kitano et al. [65] demonstrated that about 10% of human uORFs are generated and regulated by TEs localized in the 5′-UTR of mRNAs. While previous studies have suggested that retrotransposons act as translational regulators, the role of DNA transposons in influencing protein-host translation is still not clear. Miniature inverted-repeat transposable elements (MITEs) are sub-family of DNA transposons widely distributed in plant and animal genomes. It has been reported that their localization in the 3′-UTRs of rice mRNAs can exert a regulatory function via a translational repression mechanism. One example is the Ghd2 gene, a member of the CCT (CONSTANS (CO), CO-LIKE and TIMING OF CAB1) gene family in rice that regulates important agronomic traits, such as grain number, plant height, and heading date [66]. The mechanism underlying the translational repression of Ghd2 by the MITEs results on the Dicer-like 3a (OsDCL3a) pathway which can generate shortened mRNA lacking a poly-A tail by processing the MITE nascent transcripts [66]. However, which mRNA translation phase is blocked and how the mRNA translation is repressed by MITEs remains unknown [66]. When in the coding region of genes, TEs can be implicated in generating new alternative mRNA splicing isoforms. This can be seen as an intermediate step in the rise of new genes during evolution. For example, the mammalian thymopoietin (TMPO) and zinc finger protein 451 (ZNF451) genes both code for splice isoforms containing lamina-associated polypeptide 2alpha (LAP2alpha) domains, which are related to the first ORF from a Dictyostelium intermediate repeat sequence 1 (DIRS1)-like retrotransposon. Both mRNAs produce the canonical protein and a new non-canonical protein isoform. Specifically, the LAP2alpha specific isoform of TMPO (LAP2a) was co-opted during evolution to support a new and important role in the cell [67].

The evolutionary insertion of TEs in gene coding regions has also produced chimeras in a process called domestication. The activity regulated cytoskeleton associated Protein (Arc) is a good example of retrotransposon domestication, with much of the evidence suggesting it is derived from a vertebrate lineage of Ty3/gypsy retrotransposons [68]. In particular, Arc is a cellular immediate-early gene required for learning and memory, and whose mRNA is localized at the synaptic junction. Interestingly, the regulation of Arc mRNA resembles that of viral RNA, as Arc contains an internal ribosomal entry site that enables cap-independent translation [69]. Crystallography has shown that protein structure of the Arc subdomains forms a bi-lobar architecture similar to the capsid domain of the human immunodeficiency virus gag protein [69]. These findings suggest that evolution have repurposed Gag containing elements to mediate intercellular communication in the nervous system [69].

## 3. Conclusions

We have provided an overview of several new findings that corroborate the ever more evident active role of TEs in genome function, emphasizing their effects on gene expression regulation. While in many examples, it is evident that TE co-option has been positively selected for during evolution, with TE sequences becoming fixed in the genome, other examples have argued the possibility that more complex molecular mechanisms have evolved to control mobilization and co-option in the same individual during specific periods of embryonic development or under specific environmental stimuli. Although interesting questions about these mechanisms remain unraveled, it is evident that TEs represent a powerful arsenal in the many layers of gene expression regulation.
